# Cold-Induced Vasodilation during Single Digit Immersion in 0°C and 8°C Water in Men and Women

**DOI:** 10.1371/journal.pone.0122592

**Published:** 2015-04-17

**Authors:** Christopher James Tyler, Tom Reeve, Stephen S. Cheung

**Affiliations:** 1 Department of Life Sciences, University of Roehampton, London, United Kingdom; 2 Department of Kinesiology, Brock University, Ontario, Canada; The University of Tokyo, JAPAN

## Abstract

The present study compared the thermal responses of the finger to 0 and 8°C water immersion, two commonly used temperatures for cold-induced vasodilation (CIVD) research. On two separate and counterbalanced occasions 15 male and 15 female participants immersed their index finger in 20°C water for 5 min followed by either 0 or 8°C water for 30 min. Skin temperature, cardiovascular and perceptual data were recorded. Secondary analyses were performed between sexes and comparing 0.5, 1 and 4°C CIVD amplitude thresholds. With a 0.5°C threshold, CIVD waves were more prevalent in 8°C (2 (1 – 3) than in 0°C (1.5 (0 – 3)), but the amplitude was lower (4.0 ± 2.3 v 9.2 ± 4.0°C). Mean, minimum and maximum finger temperatures were lower in 0°C during the 30 min immersion, and CIVD onset and peak time occurred later in 0°C. Thermal sensation was lower and pain sensation was higher in 0°C. There were no differences between males and females in any of the physiological or CIVD data with the exception of SBP, which was higher in males. Females reported feeling higher thermal sensations in 8°C and lower pain sensations in 0°C and 8°C compared to males. Fewer CIVD responses were observed when using a 4°C (1 (0 – 3)) threshold to quantify a CIVD wave compared to using a 1°C (2 (0 – 3)) or 0.5°C (2 (0 – 3)) amplitude. In conclusion, both 0 and 8 °C can elicit CIVD but 8°C may be more suitable when looking to optimise the number of CIVD waves while minimising participant discomfort. The CIVD response to water immersion does not appear to be influenced by sex. Researchers should consider the amplitude threshold that was used to determine a CIVD wave when interpreting previous data.

## Introduction

Exposure to cold environments induces a sympathetically-mediated initial vasoconstriction of the peripheral blood vessels, with a subsequent fluctuation in skin blood flow and skin temperature with continued cold exposure—a phenomenon termed cold-induced vasodilation (CIVD) [1,2]. Despite >80 years of data collection, consolidating the CIVD literature into a coherent overall framework has been impeded by methodological differences between studies [3]. The lack of standardisation of factors such as water temperature, immersion site, immersion depth, ambient temperature, participant characteristics, pre-test cold exposure, CIVD classification amplitude and duration thresholds have led to discrepancies in research conclusions about CIVD and has hindered the identification of the underlying mechanisms and potential countermeasures. Since Lewis [2] reported that CIVD can occur in water temperatures below 15^°^C, water temperatures ranging from 0–15°C have been utilized to elicit and study CIVD. 0°C is a commonly used water temperature, especially out of the laboratory environment, due to the relative ease of creating it using a combination of water and snow/ice [4,5]; however, prolonged immersion of the finger in very cold water is often painful, and immersion of the whole hand or foot is often intolerable beyond a few minutes; indeed, the cold-pressor test used specifically for eliciting extreme pain sensations comprises of whole-hand immersion into cold water [6].

Data from two independent investigations suggested that the frequency of the CIVD response may be higher when using water at 8°C (frequency = 98.5%) compared to 4–5°C (70%) [7,8] but comparing independently derived CIVD data is fraught with problems due to the large inter-individual variability of the CIVD response [5]. A recent direct comparison reported that there was no difference in CIVD frequency between 5 and 8^°^C immersion (100% response; amplitudes of 4.2 ± 2.6°C and 3.4 ± 2.0°C, respectively) but that both temperatures elicited more frequent and stronger CIVD responses than immersion in warmer water (10°C: 87.5% response and 2.1 ± 1.6°C; 15°C 37.5% response and 2.8 ± 2.0°C) [9]. These frequency data differ from the independently obtained data [7,8] and interestingly, the differences reported above were observed despite all three investigations using the same liberal amplitude threshold of +0.5°C. More conservative thresholds such as +1°C [10,11] and +4°C [12] have been used to exclude minor fluctuations, and it is unsurprising to see that the percentage of responses reported is lower in these studies than in those using +0.5°C.

As with most of the physiological literature [13] male participants dominate the CIVD literature; however, mixed-sex populations are not uncommon [8,11,12]. Despite the mixed-sex groups very little is known about whether the CIVD response differs between males and females. Females have a more pronounced reduction in skin temperature and peripheral blood flow when exposed to cold than males [14,15] and although the reason is not fully clear it does not appear to be due to differences in plasma concentrations of oestrogen or progesterone [15]. CIVD data for male versus female comparisons are sparse but it has been suggested that, despite the enhanced vascular reactivity observed in female participants exposed to cold [15], temperature profiles of CIVD do not differ between the sexes [1]. This is yet to be experimentally established.

CIVD research has utilized single-digit exposure for methodological ease in challenging situations and whole extremity exposure for increased ecological validity (i.e., single digit exposure to cold in field settings is rare compared to whole-extremity exposure but if you wish to explore the CIVD response in extreme environments (e.g. polar expeditions) exposing the whole hand is unnecessarily risky). Recently the effect of immersing the entire hand in water 5°C and warmer [9] has been investigated; however such water temperatures can be problematic to obtain in field settings where 0°C is more commonly used and so, while this investigation has made a valuable initial contribution to comparing the CIVD response observed in different water temperatures the comparative data cannot be accurately transferred to such field settings. Therefore, the aim of the present study was to compare the CIVD, temperature, cardiovascular and perceptual responses to single finger immersion in 0°C and 8°C water in males and females. By extrapolating the data from Mekjavic et al. [9] it was hypothesised that there would be no difference in the frequency of CIVD responses between trials but that there would be a greater amplitude and higher subjective ratings of pain and discomfort in 0°C compared to 8°C. Sex was expected to have no effect on the CIVD response [1] but it was hypothesised that lower skin temperatures would be observed in female participants [14,15].

## Materials and Methods

### Ethical statement

All participants provided oral and written informed consent prior to completing a health screening. The study was approved by the University of Roehampton’s Ethical Advisory Committee.

### Participants

Thirty participants (15 male and 15 female) (mean ± SD age of 27.8 ± 7.3 years, stature of 174.0 ± 10.4 cm and body mass of 73.9 ± 14.3 kg) volunteered for this study. The mean (± SD) age, stature and body mass of the 15 male and 15 female participants were 28.5 ± 5.8 v 27.1 ± 8.6 years (t_28_ = 0.53, p = 0.60), 181.3 ± 7.1 v 166.7 ± 7.8 cm (t_28_ = 5.4, p < 0.001) and 81.8 ± 12.7 v 66.0 ± 11.5 kg (t_28_ = 3.6, p = 0.001) respectively. One participant was left handed, all were non-smokers, and none suffered from Raynaud’s syndrome or had previously experienced a cold injury.

### Protocol

Participants attended the laboratory on two occasions separated by 6 ± 2 days (range: 3–7 days). The ambient conditions in the laboratory were similar on the days of each visit (ambient temperature 22.6 ± 1.8 v 22.8 ± 1.6°C [t_29_ = 0.69, p = 0.50]; ambient humidity 36 ± 9 v 35 ± 7% [t_29_ = 0.60, p = 0.56]). All trials took place during the UK autumn and winter months (November—February) and at the same time of day for each participant. Participants wore their usual attire (trainer or shoes, socks, trousers or jeans and a t-shirt or shirt) on each occasion and were instructed to wear the same outfit on each occasion. Participants entered the laboratory and sat for 5 min while measurement equipment was attached before immersing the index finger of their non-dominant hand up to the proximal knuckle in a water bath (Clifton NE4-D, Nickel Electro Ltd., Somerset, UK) at 20°C for 5 min to standardise initial skin temperature. 20°C was used due to equipment constraints and although a warmer temperature would have been preferable 20°C did ensure a consistent starting temperature for all participants in both trials. After 5 min measurements were recorded, the participant removed their finger from the water bath and immediately immersed it in an adjacent polystyrene ice box filled with water cooled to either 0.1 ± 0.2°C or 8.1 ± 0.3°C for 30 min. Water was circulated and its temperature was recorded at 1 min intervals. The water temperature was adjusted using refrigerated water and crushed ice (to decrease) and non-refrigerated water (to increase) when appropriate. After the 30 min immersion period participants removed their finger and rested it on the table in ambient conditions for 5 min. The trial order was randomised and counterbalanced.

### Measurements

A skin thermistor (THERM 37904, Viamed Ltd, UK) was used to measure the temperature of the skin lateral to the nail bed of the immersed index finger. The thermistor was attached using a single layer of transparent dressing (Transpore White, 3M Health Care, USA) and was connected to a digital reader (Thermistor Thermometer 5831, DigiTec Corporation, USA). Skin temperature was recorded at 1 min intervals during the immersion (0–30 min) and recovery phase (30–35 min).

Heart rate (HR) (Polar Electro Oy, Kempele, Finland) was recorded at baseline and at 5 min intervals throughout the 35 min experimental period and expressed as %HR max [16] due to the age range of the participants. Arterial pressure was measured using a digital sphygmometer (UA767 Digital blood pressure monitor, A&D Company Ltd., Tokyo, Japan) at the end of the 5 min 20°C immersion and again at the end of the 30 min experimental (0 or 8°C) immersion. Arterial pressure was recorded while the participant was seated and from the non-immersed arm. Mean arterial pressure (MAP) was calculated using the formula ((DAP*2) + SAP)/3 (where DAP = diastolic arterial pressure and SAP = systolic arterial pressure).

Participants were asked to rate their thermal sensation (TS) for the immersed finger using a nine-point scale ranging from 0 (unbearably cold) to 8 (unbearably hot) with 4 as comfortable (neutral) [17] every 5 min. Pain sensation (PS) was also assessed at 5 min intervals using a Numeric Pain Distress scale ranging from 0 (no pain) to 10 (unbearable pain) as per recent CIVD research [11].

### Statistical analyses

Parametric data are presented as mean ± SD while non-parametric data are presented as median and range. Two-way (water temperature*time and water temperature*threshold amplitude) repeated measures mixed ANOVA and paired t-tests were performed on parametric data and Cohen’s *d* effect sizes were calculated for pairwise differences. Bonferroni corrections for repeated analyses were performed. Analyses were performed for the complete data set and after making list-wise exclusions when data were missing (e.g. when not all participants experienced a CIVD response). Frequency data were compared using one-way Chi square tests. Friedman’s ANOVA and Wilcoxon signed-rank tests were run and effect sizes (*r*) calculated for non-parametric data. Cohen's classification of effect size magnitude was used for interpretation, whereby *d*/*r* < 0.19 = negligible effect; *d*/*r* = 0.20–0.49 = small effect; *d*/*r* = 0.50–0.79 = moderate effect and *d*/*r* > 0.8 = large effect [18]. Pearson’s correlation analyses were used to investigate the relationships between finger temperatures and water temperature while Kendall’s tau (τ) correlation analyses were run to investigate the relationship between perceptual and temperature variables. Kendall’s tau was used due to the high number of tied ranks. CIVD parameters were identified by two researchers independently before data were compared. Discrepancies were discussed and adjudicated by a third individual if required. CIVD parameters were determined as follows [1,9]:

1. Number of waves (N): to be characterised as a CIVD response an increase in temperature of > 0.5°C lasting for > 3 min must have been observed.
2. Minimum temperature (T_min_): the lowest temperature prior to the onset of CIVD
3. Maximum temperature (T_max_): the highest temperature during the CIVD
4. Mean temperature (T_mean_): the mean temperature during the water immersion period (5–30 min)
5. Temperature amplitude (ΔT): T_max_—T_min_
6. Peak time (Δt_peak_): time interval between T_min_ and T_max_
7. Onset time (Δt_onset_): time between immersion and T_min_

The rate of rise was calculated taking into account the temperature at the beginning (30 min) and end (35 min) of the recovery phase post-immersion. In order to facilitate comparison with other data secondary analysis was also performed on the effect of using 1°C [10,11] and 4°C [19] as threshold amplitudes compared to 0.5°C. All data were collected from all participants (N = 30) with the exception of BP data which was collected from 28 participants (15 male and 13 female). Significance was accepted at p < 0.05. Raw data are contained in S1 Dataset).

## Results

### Finger temperatures

There was no difference in skin temperature pre-immersion between trials (20.1 ± 0.6 v 20.4 ± 1.2°C; t_29_ = 1.7, p = 0.10). Skin temperature was higher in 0°C following the 5 min recovery period (35 min) (26.4 ± 3.4°C v 24.3 ± 5.5°C; t_29_ = 3.0, p = 0.005) and the rate of rise during recovery was greater in 0°C compared to 8°C (4.0 ± 0.6 v 2.7 ± 0.9°C^.^min^-1^; t_29_ = 7.4, p < 0.001, *d* = 1.3). T_mean_ was lower in 0°C compared to 8°C (4.9 ± 2.8 v 11.0 ± 1.9°C; t_29_ = 19.1, p < 0.001, *d* = 1.6). There were no differences between sexes for pre-immersion skin temperature (t_58_ = 0.98, p = 0.33), skin temperature at 35 min (t_58_ = 1.5, p = 0.15), rate of rise during recovery (t_58_ = 1.6, p = 0.11) or T_mean_ (t_58_ = 0.73, p = 0.47). There was a significant relationship between water temperature and T_mean_ (*r* = 0.79, p < 0.001), T_min_ (*r* = 0.99, p < 0.001) and T_max_ (*r* = 0.42, p < 0.001).

### CIVD responses (+ 0.5°C amplitude)

Most of the CIVD data are summarised in Table 1. There were 103 CIVD responses overall. There was no difference between the total number of responses observed for male (51) or female (52) participants (χ^2^_1_ = 0.0, p > 0.99) nor were there any differences between sexes for 0°C (male = 21; female = 25; χ^2^_1_ = 0.2, p = 0.65) or 8°C trials (male = 30; female = 27; χ^2^_1_ = 0.1, p = 0.78)). The median number of CIVD responses was greater in 8°C compared to 0°C trials (2 (1–3) v 1.5 (0–3); *z* = -2.1, p = 0.03, *r* = -0.40) but did not differ between sexes (Male = 2 (0–3); female = 2 (1–3); z = -0.10, p = 0.92, *r* = -0.01).

**Table 1:** Cold-induced vasodilation data during 0 and 8°C water immersion.

		All data		List-wise excluded analysis	
		0°C	8°C	0°C	8°C
CIVD1					
Responders	N (%)	29 (97)	30 (100)	29 (97)	97)
- Male		14 (93)	15 (100)	14 (93)	14 (93)
- Female		15 (100)	15 (100)	15 (100)	15 (100)
Onset time (Δt_onset_)	min	9.0 ± 2.6	5.9 ± 2.6	9.0 ± 2.6^***^	± 2.5
- Male		8.5 ± 3.5	5.7 ± 2.2	8.6 ± 3.6	5.4 ± 1.9
- Female		8.7 ± 2.6	6.0 ± 3.1	8.7 ± 2.6	6.0 ± 3.1
Peak time (Δt_peak_)	min	9.5 ± 4.0	6.0 ± 2.4	9.5 ± 4.0^***^	5.9 ± 2.3
- Male		7.5 ± 3.6	5.9 ± 2.2	7.6 ± 3.7	5.6 ± 1.9
- Female		10.2 ± 4.0	6.1 ± 2.7	10.2 ± 4.0	6.1 ± 2.7
Minimum temperature (T_min_)	°C	0.6 ± 0.5	8.8 ± 0.9	0.6 ± 0.5^***^	± 0.9
- Male		0.4 ± 0.3	8.5 ± 0.7	0.4 ± 0.3	8.5 ± 0.7
- Female		0.7 ± 0.7	8.9 ± 0.9	0.7 ± 0.7	8.9 ± 0.9
Maximum temperature (T_max_)	°C	9.8 ± 4.2	12.8 ± 2.5	9.8 ± 4.2^***^	12.9 ± 2.5
- Male		9.8 ± 4.6	14.2 ± 3.5	10.4 ± 4.1	14.5 ± 3.4
- Female		9.7 ± 4.5	12.1 ± 1.7	9.7 ± 4.5	12.1 ± 1.7
Amplitude (ΔT)	°C	9.2 ± 4.0	4.0 ± 2.3	9.2 ± 4.0^***^	± 2.3
- Male		9.2 ± 4.4	5.1 ± 2.6	9.1 ± 4.6	5.4 ± 2.5
- Female		8.7 ± 4.4	2.8 ± 1.2	8.7 ± 4.4	2.8 ± 1.2
CIVD2					
Responders	N (%)	15 (50)	21 (70)	10	10
- Male		6 (20)	12 (40)	4 (13)	4 (13)
- Female		9 (30)	9 (30)	6 (20)	6 (20)
Onset time (Δt_onset_)	min	21.1 ± 4.1	17.7 ± 4.6	21.1 ± 4.1^*^	17.7 ± 4.6
Peak time (Δt_peak_)	min	4.9 ± 2.1	4.4 ± 1.4	4.9 ± 2.1	4.4 ± 1.4
Minimum temperature (T_min_)	°C	6.4 ± 4.2	10.7 ± 2.0	6.4 ± 4.1^**^	10.7 ± 2.0
Maximum temperature (T_max_)	°C	10.2 ±4.8	13.4 ± 3.1	10.2 ± 4.8	13.4 ± 3.1
Amplitude (ΔT)	°C	3.8 ± 2.8	2.8 ± 1.8	3.8 ± 2.8	2.8 ± 1.8
CIVD3					
Responders	N (%)	2 (7)	6 (20)	0	0
- Male		1 (3)	3 (10)		
- Female		1 (3)	3 (10)		
Onset time (Δt_onset_)	min	22.5 ± 0.7	22.2 ± 1.9		
Peak time (Δt_peak_)	min	4.0 ± 1.4	5.3 ± 2.1		
Minimum temperature (T_min_)	°C	8.9 ± 8.2	10.5 ± 1.0		
Maximum temperature (T_max_)	°C	12.0 ± 10.0	12.4 ± 2.1		
Amplitude (ΔT)	°C	3.1 ± 1.8	1.9 ± 1.5		

^*^ p < 0.05;

^**^ p < 0.01;

^***^ p < 0.001

**Fig 1:**
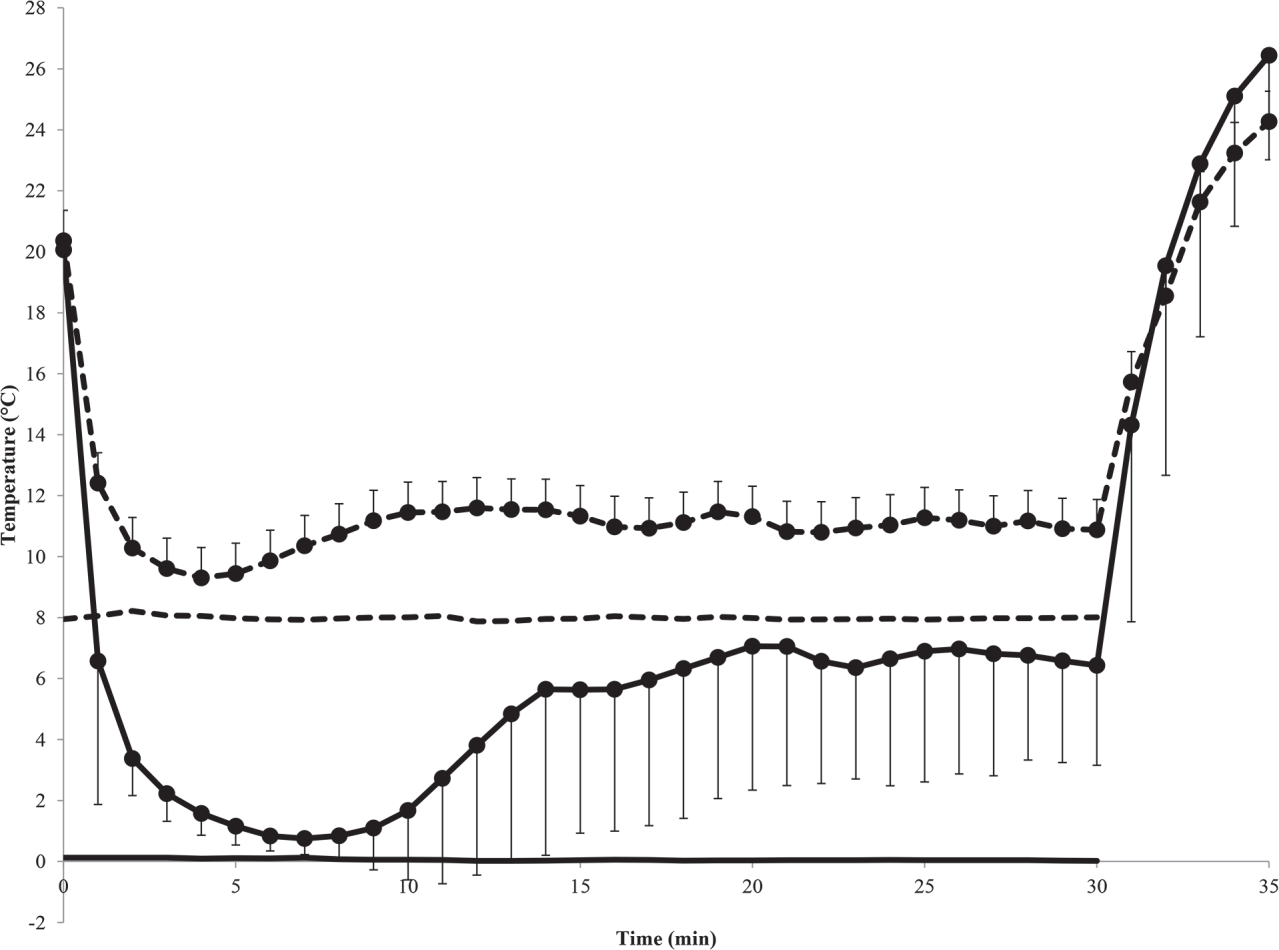
Mean (± SD) finger temperature during immersion and recovery and mean water temperature during immersion. Dashed line = 8°C trial; solid line = 0°C trial. Marker = finger temperature; no marker = water temperature.

Pairwise analyses of the 1^st^ and 2^nd^ CIVD responses revealed that T_min_ was lower in 0°C for both waves; T_max_ was lower in 0°C during the 1^st^ wave; ΔT was lower in 8°C during the 1^st^ wave; Δt_onset_ was later in 0°C for both waves and Δt_peak_ was longer in 0°C during the 1^st^ wave (Table 1; analysis column). Eight separate participants had a 3^rd^ CIVD response in 0°C (n = 2) and 8°C (n = 6). There were no interaction effects (p = 0.12–0.74) or differences between sexes (p = 0.07–0.68) for any variable measured during the 1^st^ CIVD wave (p > 0.05). Finger and water temperatures recorded during immersion and recovery are shown in Fig 1.

### Comparison of +0.5, 1 and 4°C amplitude thresholds

**Fig 2:**
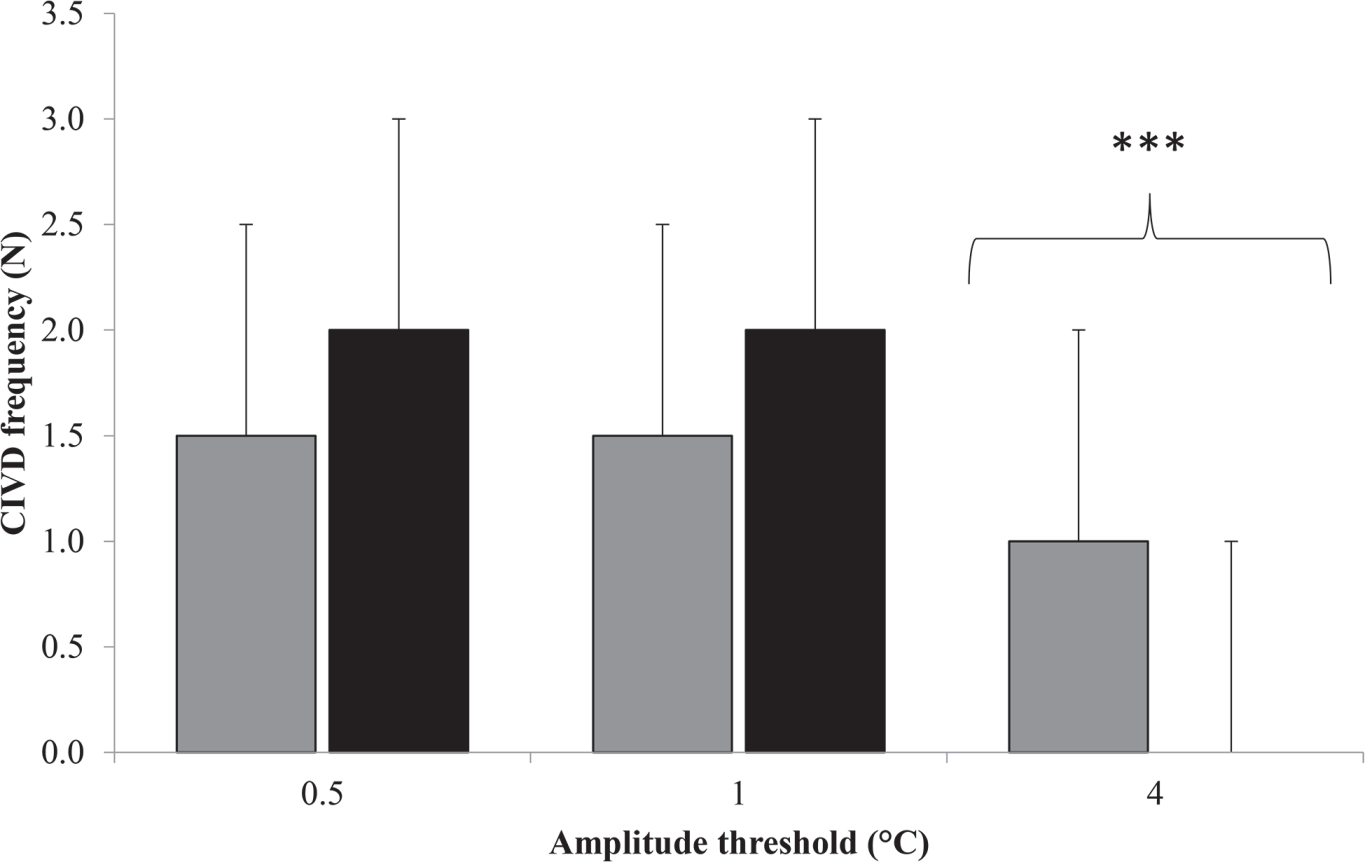
Median (range) frequency (N) when using amplitude thresholds of 0.5, 1 and 4°C. Grey fill = 0°C trial; Black fill = 8°C; ^***^ = significantly lower than 0.5°C and 1°C (p < 0.001).

Fig 2 displays the median (range) frequency when using amplitude thresholds of 0.5, 1 and 4°C. There was a significant main effect for amplitude threshold (χ^2^_2_ = 65, p < 0.001). Pairwise analyses revealed that fewer CIVD responses were observed when using 4°C (Mdn = 1 (0–3)) compared to 0.5°C (Mdn = 2 (0–3); *z* = -5.2, p < 0.001, *r* = -0.67) and 1°C (Mdn = 2 (0–3); *z* = -5.2, p < 0.001, *r* = -0.67). There was no difference between 0.5 and 1°C compared to 0.5°C (z = -1.0, p = 0.6, *r* = -0.14).

### Cardiovascular responses

The was no difference between trials for SAP (115 + 11 v 113 ± 10 mmHg; main effect trial: F_1,26_ = 1.5, p = 0.23; time: F_1,26_ = 0.7, p = 0.41; trial x time interaction: F_1,26_ = 0.6, p = 0.82), DAP (71 + 10 v 69 ± 8 mmHg; main effect trial: F_1,26_ = 2.8, p = 0.11; time: F_1,26_ = 0.14, p = 0.71; trial x time interaction: F_1,26_ = 0.2, p = 0.70) or MAP (86 + 10 v 84 ± 8 mmHg; main effect trial: F_1,26_ = 2.8, p = 0.11; time: F_1,26_ = 0.54, p = 0.47; trial x time interaction: F_1,26_ = 0.2, p = 0.68) in 0 and 8°C trials respectively. Sex had no effect on DAP (71 ± 8 v 70 ± 9; p = 0.77) or MAP (86 ± 7 v 83 ± 8; p = 0.19) but SAP was higher in male participants (118 ± 7 v 109 ± 8; p = 0.002). There was no difference between trials for %HR_max_ (0°C = 38 ± 5%; 8°C = 39 ± 5%; main effect: F_1,28_ = 1.1, p = 0.30) nor was there any interaction effects between trial, time and sex (p = 0.22–0.64). %HR_max_ changed over time (F_3.5,98.5_ = 4.5, p < 0.001) and was higher at 5 and 10 min compared to 25 (p = 0.024 and < 0.01), 30 (p = 0.01 and 0.01) and 35 min (p < 0.01 and 0.001).

### Perceptual responses

**Fig 3:**
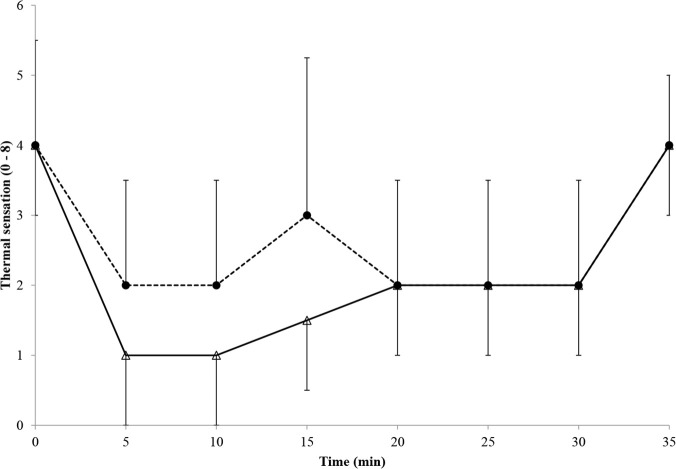
Median (range) thermal sensation during immersion and recovery. Dashed line and filled circles = 8°C trial; solid line and open triangles = 0°C trial.

**Fig 4:**
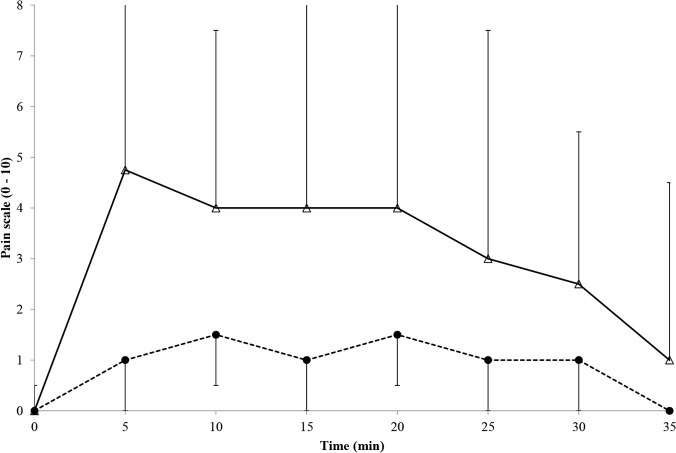
Median (range) pain sensation during immersion and recovery. Dashed line = 8°C trial; solid line = 0°C trial.

Perceptual data are shown in Figs 3 and 4. TS was lower (Mdn = 2 (0–5) v 3 (0.5–5); *z* = -7.4, p < 0.001, *r* = -1.35) and PS was higher (Mdn = 3 (0–9) v 1 (0–7); *z* = -10.8, p < 0.001, *r* = -2.0) in 0°C compared to 8°C. Perceptual data changed over time (TS: χ^2^_7_ = 247, p < 0.001; PS: χ^2^_7_ = 226, p < 0.001)). For TS all time-point comparisons with the exception of 0 v 35 min (p = 0.05); 15 v 10 (p = 0.06), 20 (p = 0.31), 25 min (p = 0.33) and 30 min (p = 0.06); 20 v 25 (p = 0.85) and 30 min (p = 0.24); and 25 v 30 min (p = 0.18) were significantly different from one another (p < 0.001–0.01). For PS all time-point comparisons with the exception of 5 v 10 min (p = 0.62); 15 v 10 (p = 0.06) and 20 min (p = 0.23); and 20 v 25 min (p = 0.18) were significantly different from one another (exact p < 0.001–0.04). TS did not differ between sexes in 0°C (Mdn = 2 (0–5) for both; z = -0.07, p = 0.95) but was higher in 8°C in female (3.0 (0.5–5) compared to male (2.5 (1–5) participants (z = -2.1, p = 0.03, *r* = -0.39). PS was lower in female participants in 0°C (3.0 (0–9) v 3.8 (0–9); z = -2.9, p = 0.004, *r* = -0.53) and 8°C (0.0 (0–5) v 1.8 (0–7); z = -5.3, p < 0.001, *r* = -0.97). There was a high-strength, positive correlation between TS and finger temperature (τ = 0.55, p < 0.001) and high-strength, negative correlations between PS and finger temperature (τ = -0.51, p < 0.001) and between PS and TS (τ = -0.63, p < 0.001).

## Discussion

The present study sought to compare two water temperatures commonly employed in lab- and field-based CIVD research, with the aim of determining the validity of comparing data across studies. Our data provides further evidence that the CIVD response is dependent upon the temperature of the water used for immersion and also that CIVD data are influenced by the threshold temperature used to quantify a response. Our data also suggests that the CIVD response is similar between males and females which is in line with previous, observational data [1] but unlike previous data [14,15] lower skin temperatures were not observed in female participants in the current study. Overall, while methodologically more challenging in the field, water temperatures of ~ 8°C may be optimal for investigating the CIVD response by maximizing CIVD events while minimizing systemic cardiovascular changes along with perceptual discomfort and pain amongst participants.

Both the empirical categorization and the exact mechanisms underlying the CIVD response remains unclear >80 years following their initial documentation [2]. Contributing to the difficulty in consolidating the literature has been methodological differences across independently conducted studies, and the need for consistency across testing protocols has recently been highlighted [3]. The standardisation of variables such as water temperature, immersion site and depth, CIVD classification amplitudes and durations would benefit future exploration of the CIVD mechanisms while direct comparisons of the various methodological differences previously used (e.g. amplitude thresholds used and participant characteristics) would aid the comparison between, and interpretation of, the existing body of literature. Both 0°C (29/30 or 97%) and 8^°^C (30/30 or 100%) finger immersion were capable of eliciting at least one CIVD response for all bar one of the participants. Mekjavic et al. [9] reported greater CIVD frequency in 8^°^C compared to warmer (10 and 15°C) water, and that there was a *“tendency*, *albeit not statistically significant (p = 0*.*06)”* (p 18) for more CIVD waves to occur in 8°C compared to 5°C trials. Thus, 8°C water may form an optimal temperature for CIVD frequency. The frequencies observed in the present study were lower than those reported by Mekjavic et al. [9] in their cooler trials (8°C ~3 waves; 5°C = ~2.5 waves) but comparable to their warmer trials (10°C = ~2 waves; 15°C ~1 CIVD wave). Such variability between studies highlights the large degree of heterogeneity between individuals in the CIVD response [5] and it is possible that the difference in frequency between studies was observed because our larger population and lower sampling frequency reduced the overall group variation. Furthermore, some differences in the nature and magnitude of the CIVD response did occur between 0^°^C and 8^°^C immersion. Specifically, more participants had a second CIVD response in 8°C trials (21 v 15/30), likely as the initial CIVD response was delayed (later Δt_onset_), more pronounced (greater Δt_peak_) and longer in duration (ΔT) in 0°C trials.

None of the CIVD data differed between male and female participants, which supports previous suggestions that the CIVD hunting response is similar between males and females [1] but conflicts with data that females have lower skin temperatures during cold-water immersion [14]. Bartelink et al. [14] investigated the effect of hormonal status on skin vascular activity and reported that finger skin temperature was higher in male participants compared to female participants; however, they used a warmer temperature (15°C) and the difference in water temperature may explain the difference in data because the peripheral blood flow response to cold exposure appears to be dependent upon the extent of the cold exposure.

While the greater magnitude and duration of CIVD responses might suggest a preference for 0^°^C immersion, perceptual sensations and participant tolerance argue for the adoption of 8°C immersion. Warmer temperatures are perceived as less painful by participants [9,20] and, in the present study, participant discomfort was greater (as demonstrated by a colder thermal sensation and higher pain sensation) in the 0°C trial and this was more pronounced in male participants. Unpleasant thermal sensations are related to lower temperatures, and the present study demonstrated a strong positive correlation between thermal sensation and finger temperature, along with strong negative correlations between pain sensation and finger temperature and between pain sensation and thermal sensation. These data, in combination with the frequency responses being similar between 8^°^C and 0^°^C, suggest that 8^°^C immersion can form an optimal balance between CIVD wave frequency and participant tolerance. Sendowski et al. [21] reported that hand immersion results in greater ratings of pain than finger immersion when water temperature is the same, thus it is prudent to suggest that the differences observed in the present study would be exacerbated if whole-hand immersion was used.

Beyond thermal and perceptual perturbations, immersion temperature may influence cardiovascular responses [9,21]. Mekjavic et al. [9] reported that HR was unaffected by water temperature but that MAP was elevated in 5 and 8°C compared to 10 and 15°C, while Kregel et al.[20] reported that HR and arterial pressure changes to whole-hand immersion were dependent upon the temperature used for immersion. Data from the current investigation showed that there was no difference in the MAP response to 0 and 8°C immersion; however, it is worth noting that data have demonstrated that finger immersion can elevate MAP in the 4 minutes immediately following immersion [9,21]. Unfortunately arterial pressure data were not acquired during immersion in the present study. Previous data have suggested that finger immersion does not reduce HR and that whole-hand immersion is required to observe reductions [9,21] and this is supported by data from the present study. HR was elevated at 5 and 10 minutes by the immersion but data at 0 min was comparable to that recorded from 15 minutes onwards.

Some of the differences between investigations can be attributed to differences in the amplitude used to characterise a response. As with much of the CIVD literature there is a lack of standardisation regarding the magnitude of temperature increase used to identify a CIVD wave and, as a result, temperature thresholds ranging from 0.5°C [22] to 4°C [12] have been used. Not surprisingly, the present study highlights that frequency is higher when using the most commonly used threshold (0.5°C) compared to the more conservative alternative (4°C); however, the data also highlights that different thresholds result in relatively consistent frequencies being reported when standardised classification criteria are used to define CIVD parameters. Due to the fact that 0.5°C is the most commonly used amplitude it is suggested that future research uses this threshold but researchers should take into account the difference in frequency between amplitude thresholds when interpreting data from previous published manuscripts.

In conclusion, the combined physiological and perceptual data suggest that 8°C water immersion may be an optimal temperature for CIVD investigations because it can be easily created in a laboratory, induces universal CIVD reactions and is better tolerated than colder temperatures. 0°C can be used in field settings where it may be impractical/impossible to create an 8°C condition. From a safety perspective it is worth noting that the rate at which finger temperature recovers from cold immersion is greater the colder the water used [23] and so 0°C single-digit immersion is safe to use if a warmer temperature is not feasible. There appears to be no difference between males and females with regards to CIVD responses.

## Limitations

As previously mentioned, there are many methodological inconsistencies in the CIVD literature. While the data from this manuscript goes someway to providing data to quantify the effect of water temperature, amplitude threshold and sex differences there are other methodological issues of note such as the effects of age, habituation to cold and the menstrual cycle. The CIVD response occurs later and to less an extent in older populations [24] and earlier in long-term acclimatised individuals [25]. This highlights that the age of the individual and the extent of their prior exposure to cold can affect the CIVD response observed; however, many laboratory studies have reported no improvement in the CIVD response following short-term acclimation [3]. None of our participants had undergone experimental cold acclimation but due to testing taking place during the colder months of the year some acclimatisation may have occurred. The mean age of the participants in this study (27.8 ± 7.3 years) is likely to be lower than that of the majority of workers and explorers who may be exposed to extreme cold. CIVD responses are more pronounced and earlier in younger individuals [24] and so the age of the participants needs to be considered when comparing data from the current study to that from previous and future investigations. The present study offers support for previous suggestions that, despite the enhanced vascular reactivity observed in female participants exposed to cold [15], temperature profiles of CIVD do not appear to differ between the sexes [1] but the phase of the menstrual cycle was not controlled for. Previous data suggest that there is no relationship between skin blood flow and plasma concentrations of oestrogen or progesterone [15]; however, due to the paucity of data it would be prudent to investigate the effect of menstrual cycle phase on the CIVD response and/or control for it in subsequent sex comparison investigations. Finally, blood flow responses to cold exposure are influenced by clothing and, although participants were instructed to wear identical clothing on each visit, it is possible that slight differences in clothing existed. Future investigations should consider standardising the clothing worn to control for this potential source of experimental noise.

## Practical Implications

Data from the present study suggest that 8°C water immersion may be an optimal temperature for CIVD investigations because it can be easily created in a laboratory, induces universal CIVD reactions and is better tolerated than colder temperatures although 00078C can still be used in field settings where it may be impractical/impossible to create an 8°C condition. It is recommended that an amplitude threshold of 0.5°C is used in future research. The data suggests that mixed-sex groups can be used to investigate CIVD due to the lack of differences between men and women but this are still requires further research.

## Supporting Information

S1 Dataset (XLSX)
