# Correction: Cold-Induced Vasodilation during Single Digit Immersion in 0°C and 8°C Water in Men and Women

**DOI:** 10.1371/journal.pone.0275188

**Published:** 2022-09-20

**Authors:** Christopher James Tyler, Tom Reeve, Stephen S. Cheung

## Notice of Republication

In light of an issue identified post-publication, this article was republished on August 12, 2022 to replace S1 Dataset and remove information that was not relevant to the article. Please download this article again to view the correct version.
